# Long-Term Safety and Efficacy of Partially Absorbable Transobturator Mid-Urethral Sling in Women Aged 65 Years and Older

**DOI:** 10.3390/jcm14248838

**Published:** 2025-12-13

**Authors:** Réka Fábián-Kovács, Asnat Groutz, Jonatan Neuman, Menahem Neuman, Yoav Baruch, Ronen S. Gold

**Affiliations:** 1 Medical School, Semmelweis University, 1085 Budapest, Hungary; rekafabiankovacs@gmail.com (R.F.-K.); jonatan789@gmail.com (J.N.); 2 Urogynecology Unit, Lis Women Hospital, Tel Aviv Medical Center, Gray Faculty of Medical and Health Sciences, Tel Aviv University, Tel Aviv-Yafo 6997801, Israel; yoavi100@gmil.com (Y.B.); r_gold_il@yahoo.com (R.S.G.); 3 The Urogynecology Service, Assuta Medical Centers, Medical School, Ben Gurion University, Be’er Sheva 8410501, Israel; menahem.neuman@gmail.com

**Keywords:** mid-urethral sling, transobturator vaginal tape, elderly, partially absorbable mesh, stress urinary incontinence, long-term outcomes

## Abstract

**Objectives:** To assess the long-term safety and efficacy of the Serasis^®^ partially absorbable transobturator mid-urethral sling (MUS) in women aged ≥65 years compared to younger women. **Methods:** A retrospective comparative study of 375 consecutive women who underwent Serasis^®^ MUS for stress urinary incontinence (SUI). Patients were stratified into two age groups: 45–64 years (N = 118) and ≥65 years (N = 257), with further subdivision of the elderly cohort into 65–74 years (N = 208) and 75–84 years (N = 49). Primary outcomes included perioperative safety and long-term subjective cure rates, assessed via standardized telephone survey at a mean follow-up of 8.5 years (range, 6.8–10.9 years). **Results:** Perioperative outcomes were comparable across age groups. At 4 months postoperatively, subjective cure was achieved in 82.9% of elderly and 86.4% of younger patients. Long-term subjective cure rates were 79.6% and 85.4%, respectively (*p* = 0.27). Elderly patients experienced higher rates of postoperative voiding dysfunction and persistent overactive bladder symptoms, though subjective satisfaction remained high. Long-term mesh-related complications were infrequent across age groups. Specifically, vaginal mesh erosion requiring surgical removal occurred in three elderly patients (1.6%) and in only one younger patient (1.1%). A multivariate logistic regression analysis identified preoperative mixed urinary incontinence, BMI >30 kg/m^2^ and concomitant pelvic organ prolapse repair as independent predictors of surgical failure. Age ≥65 years was not an independent predictor of surgical failure (OR 1.3, 95% CI 0.8–2.1, *p* = 0.31). **Conclusions:** The use of a partially absorbable MUS in elderly women with SUI is a safe and effective surgical approach, associated with a significant reduction in long-term mesh-related complications.

## 1. Introduction

Stress urinary incontinence (SUI) is the most common type of urinary incontinence in women, with rates varying from 4% to over 35% among adult women [1]. The prevalence of SUI increases progressively with advancing age, particularly during midlife and the peri- and postmenopausal phases. In contrast, urgency urinary incontinence (UUI) and mixed urinary incontinence (MUI) tend to become more predominant in older populations, reflecting an age-related shift in the clinical presentation of urinary incontinence. The definitive treatment for significant SUI is surgical, whereas the mainstay of therapy for UUI is pharmacological. In cases of clinically significant MUI, a combined approach that integrates surgical and pharmacological treatment may be required [2,3,4]. The increasing global aging population has resulted in a higher number of elderly women seeking surgical interventions for SUI and MUI. Furthermore, in women presenting with both urinary incontinence and pelvic organ prolapse (POP), combined surgical intervention is often necessary. Advanced age is correlated with elevated surgical risks, a higher prevalence of comorbidities, and potentially atypical healing responses, all of which may significantly impact surgical outcomes [5,6]. This demographic trend, therefore, poses distinct challenges for clinicians regarding the selection of appropriate patients and the management of outcome expectations.

Mid-urethral slings (MUS) have become the gold standard surgical treatment for SUI, with extensive evidence supporting their efficacy and safety [2,3,4]. However, the safety and efficacy of MUS procedures in elderly patients remain controversial [7,8]. Furthermore, traditional polypropylene mesh slings, while effective, have been associated with mesh-related complications including tape erosion, chronic pelvic pain, and dyspareunia [9,10]. These concerns have led to the development of partially absorbable mesh materials designed to reduce long-term mesh-related complications while maintaining surgical efficacy. Serasis^®^ (Serag-Wiessner, Naila, Germany) is a partially absorbable MUS system featuring a bi-component mesh made from non-absorbable polypropylene and absorbable polyglycolic acid-caprolactone (PGACL). The PGACL component is absorbed within 90–120 days, creating a softer fabric structure with potentially reduced tissue trauma and mesh-related complications. Clinical data from previous studies have confirmed the safety and therapeutic effectiveness of Serasis^®^, with 92% of patients exhibiting complete resolution of SUI at 12 months postoperatively, accompanied by a low rate of mesh-related complications [11,12].

Although partially absorbable mesh systems offer potential advantages, evidence regarding their safety and long-term efficacy in elderly women remains limited. The aim of the present study was to evaluate the long-term clinical outcomes associated with the use of the Serasis^®^ partially absorbable MUS in the surgical management of SUI among elderly patients.

## 2. Methods

A cohort of 375 women who underwent Serasis^®^ inside-out transobturator MUS was analyzed. Participants were stratified into two main age groups: younger women aged 45–64 years (N = 118) and elderly women aged ≥65 years (N = 257). For further analysis, the elderly group was subdivided into those aged 65–74 years (N = 208) and 75–84 years (N = 49). Clinical preoperative and early postoperative outcomes were collected retrospectively from medical records. To complement the retrospective analysis and to ensure assessment of long-term safety and efficacy, a prospective follow-up survey was conducted in August 2025. The mean follow-up duration was 8.5 years (range, 6.8–10.9 years). Women presenting with predominant preoperative UUI, identified either through patient self-report or confirmed by urodynamic evaluation, were excluded. Patients demonstrating voiding dysfunction were likewise ineligible; this was defined as persistently elevated post-void residual urine volumes documented on repeated measurements, or urodynamic abnormalities consistent with impaired bladder emptying. In addition, women with apical POP were excluded, characterized clinically as either uterine prolapse or vaginal vault prolapse. These exclusion criteria were implemented to ensure a methodologically homogeneous study population and to minimize potential confounding factors that could influence surgical outcomes or long-term follow-up. The study protocol was approved by the Institutional Review Board (ASMC 0036-24, 15 september 2024).

All procedures were performed using the Serasis^®^ (Serag-Wiessner, Naila, Germany) partially absorbable tape. The mesh implant is composed of a dual-component material with wide pores, combining non-absorbable polypropylene and absorbable PGACL. In all cases, the inside-out transobturator technique was employed. Concomitant native-tissue repair was performed as indicated for patients with cystocele and/or rectocele. All procedures were performed under regional or general anesthesia as an outpatient or short-stay procedure.

Data on demographics, clinical characteristics, intraoperative details, and postoperative outcomes were retrospectively extracted from a computerized medical record system. Preoperative evaluation included a comprehensive history, physical examination, a cough stress test to confirm SUI, and an ultrasound assessment of post-void residual urine. The POP-Q classification system was used to assess type and severity of concomitant POP [13]. Intraoperative data included duration of surgery, estimated blood loss, and surgical complications. Early follow-up assessment was performed at 1 month and 4 months postoperatively and included physical examination, subjective cure (absence of SUI), objective cure (negative cough stress test), postoperative voiding dysfunction, overactive bladder (OAB) symptoms (either persistent or de novo), pain, dyspareunia, patient satisfaction, and mesh-related complications. Persistent OAB refers to patients with a previous preoperative diagnosis who continue to experience postoperative OAB symptoms. De novo OAB denotes patients presenting with newly recognized symptoms, without any prior diagnosis or history of the condition. Mesh-related complications were classified as vaginal erosion (mesh visible or palpable through the vaginal epithelium), erosion into the urinary tract (mesh protruding into the urethra or bladder, confirmed by cystoscopy), and pain-related complications (persistent pain or dyspareunia attributed to the MUS).

A long-term follow-up survey was conducted in August 2025. Of the initial cohort of 375 patients, 275 (73.3%) were available for the long-term follow-up assessment. The patients were interviewed using a standardized script of lower urinary tract symptoms and mesh-related complications, as well as whether they had undergone additional surgery for SUI. Patient-reported outcomes were assessed using the Satisfaction with Decision Scale for female pelvic floor disorders, ranging from 0 to 100 [14]. Cure was defined as a satisfaction score of ≥80, improvement as 61–79, and failure as ≤60.

Continuous variable distribution was evaluated using histogram and QQ plots. All variables examined demonstrated a normal distribution. Statistical analysis was performed using Student’s t-test for normally distributed data. Categorical variables were analyzed using the Chi-square test or Fisher’s exact test as appropriate. Multivariate logistic regression analysis was performed to identify independent predictors of treatment failure. Data are presented as mean ± standard deviation (SD) for continuous variables and percentages for categorical variables. Statistical significance was set at *p* < 0.05. SPSS software version 27 (IBM Corporation, Armonk, NY, USA) was used for the statistical analysis.

## 3. Results

The study cohort comprised 375 consecutive patients who underwent the Serasis^®^ MUS procedure. Of these, 118 were classified as younger women, with a mean age of 54.3 ± 5.2 years, and 257 as elderly women, with a mean age of 71.1 ± 4.2 years. Within the cohort of elderly patients, 208 (80.9%) women were between 65 and 74 years of age, with a mean age of 69.5 ± 2.8 years, while 49 (19.1%) women were aged 75 to 84 years, with a mean age of 78.2 ± 2.6 years. Long-term follow-up data were available for 275 (73.3%) patients, with a mean follow-up duration of 8.5 years (range, 6.8–10.9 years).

Table 1 presents the baseline demographic and clinical characteristics of the study cohort. Compared to younger patients, elderly patients had a significantly higher number of medical comorbidities (2.1 ± 1.4 versus 0.8 ± 0.9, *p* < 0.001). The most common comorbidities among elderly patients were hypertension (45.9%), diabetes mellitus (18.3%), and osteoporosis (12.8%). Additionally, elderly patients experienced a longer duration of preoperative SUI, averaging 5.8 ± 4.2 years, compared to 4.2 ± 3.8 years in younger patients (*p* = 0.001). The incidence of MUI was similar among elderly and younger patients (30.4% and 27.1%, respectively). Preoperative concomitant cystocele of stage ≥ 2 was found in 142 (55.3%) elderly patients and in 65 (55.1%) younger patients. Similarly, stage ≥ 2 rectocele was diagnosed in 124 (48.2%) elderly patients and 58 (49.2%) younger patients.

Intraoperative and perioperative data are presented in Table 2. The inside-out transobturator approach was employed in all cases in both age groups. There were no cases of significant intraoperative complications. The mean operative time was 26.1 ± 12.3 min in the elderly group and 24.8 ± 11.2 min in the younger group. The mean estimated blood loss was minimal in both age groups. The average hospital stay was 18.9 ± 9.7 h for elderly patients compared to 16.2 ± 8.4 h for younger patients (*p* = 0.01). Most patients in both age groups were discharged on the same day as their surgery.

Four-month clinical outcomes are presented in Table 3. Subjective cure was achieved in 213 (82.9%) elderly patients and 102 (86.4%) younger patients. Patient satisfaction scores of ≥80 were reported by 228 (88.7%) elderly patients versus 108 (91.5%) younger patients. Of the elderly patients, 4 (2.6%) reported low satisfaction scores of less than 60, versus none of the younger patients. Elderly patients experienced significantly higher rates of postoperative voiding dysfunction compared with younger patients (12.8% versus 5.9%, *p* = 0.04, respectively). Specifically, 12 (4.7%) elderly patients required catheterization for more than 2 days compared with only 2 (1.7%) younger patients. All cases were managed conservatively with complete resumption of spontaneous voiding within few days. Persistent OAB symptoms occurred significantly more frequently in elderly patients than in younger ones (18.3% versus 9.3%, *p* = 0.02, respectively). De novo OAB symptoms were similar in both age groups (8.6% versus 6.8%, *p* = 0.58, respectively). There were no cases of early mesh erosion or exposure at 4 month postoperatively.

A long-term follow-up survey was conducted in August 2025. From the initial cohort of 375 patients, long-term follow-up data were available for 275 (73.3%). The mean duration of follow-up was 8.5 years, with a range of 6.8 to 10.9 years. The overall response rates observed in the study were 75.4% (89 out of 118) for the younger cohort and 72.4% (186 out of 257) for the elderly cohort. The clinical and demographic characteristics of patients who completed long-term follow-up were comparable to those of patients lost to follow-up. Long-term follow-up characteristics and outcomes are presented in Table 4. The long-term subjective cure rates remained high in both elderly and younger patients (79.6% and 85.4%, *p* = 0.27, respectively). Patient satisfaction scores ≥80 were reported by 81 (91.0%) younger patients and 164 (88.2%) elderly patients. Of the elderly patients, 4 (2.1%) reported low satisfaction scores of less than 60, versus 3 (3.4%) of the younger patients. Persistent SUI rates were more common among elderly patients; however, this difference was not statistically significant (20.4% versus 14.6%, *p* = 0.27, respectively). Sixteen (8.6%) elderly patients and 6 (6.7%) younger patients required additional surgical intervention for SUI during the long-term follow-up period. Persistent OAB was more common among elderly patients (18.3% versus 9.0%, *p* = 0.05, respectively). The rates of de novo OAB were similar in both younger (5.6%) and elderly (8.1%) patients. Long-term mesh-related complications were infrequent across age groups. Specifically, vaginal mesh erosion requiring surgical removal occurred in three elderly patients (1.6%) and in only one younger patient (1.1%).

Multivariate logistic regression analysis identified the following independent predictors of surgical failure (defined as satisfaction rates <60%): preoperative MUI (OR 2.1, 95% CI 1.2–3.7, *p* = 0.01); BMI >30 kg/m^2^ (OR 1.8, 95% CI 1.1–3.0, *p* = 0.02); and concomitant stage 3 POP repair (OR 2.3, 95% CI 1.3–4.1, *p* = 0.004). Age ≥65 years was not an independent predictor of surgical failure (OR 1.3, 95% CI 0.8–2.1, *p* = 0.31).

## 4. Discussion

Mid-urethral slings, introduced in 1995, have become the gold standard for surgical management of SUI. A wide range of MUS devices is available, varying in insertion technique and mesh composition. Most utilize lightweight, macroporous polypropylene mesh, which offers favorable integration and durability. Common MUS techniques include retropubic tension-free vaginal tape (TVT), transobturator MUS (TOT), and single-incision mini-slings (SIS). These can be inserted using either an inside-out approach (from the vaginal incision outward) or an outside-in approach (from the skin toward the vaginal incision), depending on the device design and surgical preference. Multiple studies have confirmed MUS procedures as the preferred surgical treatment for SUI, a position supported by international clinical guidelines [2,4]. A comprehensive systematic review encompassing 175 randomized controlled trials and 21,598 participants evaluated 12-month postoperative outcomes across various surgical techniques for SUI [15]. The results demonstrated high cure rates associated with pubovaginal fascial slings (89.4%) and retropubic MUS (89.1%). Conversely, lower cure rates were observed with open colposuspension (76.6%), TOT procedures (64.1%), laparoscopic colposuspension (48.9%), SIS (39.8%), and bladder neck needle suspension (26.9%).

Although MUS procedures are widely recognized for their efficacy in treating SUI, concerns remain regarding the long-term safety of synthetic mesh. Adverse outcomes such as mesh erosion and chronic pain have prompted caution, especially in older patients who frequently present with multiple comorbidities and may be predisposed to mesh-related complications due to vaginal atrophy [9,10,16]. Moreover, data on long-term safety outcomes of MUS in elderly women remain limited and non-conclusive. Gordon et al. compared 157 elderly women aged ≥70 years with 303 younger women undergoing retropubic TVT for SUI [7]. While cure rates were similar between groups, elderly patients experienced significantly higher rates of postoperative de novo UUI (18% vs. 4%, *p* < 0.05). The investigators concluded that although retropubic TVT achieves good outcomes in elderly women, the risks of postoperative de novo UUI and age-related morbidities are increased. Groutz et al. prospectively evaluated inside-out TOT in 97 elderly (≥70 years) and 256 younger women [17]. Early and late postoperative outcomes were similar between the age groups, except for significantly more postoperative recurrent urinary tract infections among elderly women (13.7% vs. 6.2%). The incidence of persistent urodynamically confirmed overt SUI was similar in both age groups (5%), though asymptomatic urodynamic SUI was more common in elderly patients (19% vs. 3.7%, *p* < 0.05). Persistent OAB rates were comparable between elderly and younger patients (68% and 62%, respectively), while de novo OAB was significantly more common in elderly patients (11.9% vs. 4.7%, *p* < 0.05). The investigators emphasized that age alone should not preclude surgical intervention for SUI. Similarly, Stav et al. examined 1225 consecutive women with a mean 50-month follow-up, comparing 96 women aged ≥80 years with 1016 younger patients [18]. Overall subjective cure rates showed no significant difference (81% vs. 85%, *p* = 0.32); however, elderly patients experienced significantly higher rates of failed first trial of void (37% vs. 9%, *p* < 0.001). The Norwegian registry analysis by Engen et al., the largest published series with 21,832 women undergoing MUS procedures between 1998 and 2016, demonstrated that women in their sixth decade and older experienced more objective persistent postoperative SUI, and those in their seventh decade and older reported lower postoperative satisfaction [19]. OAB symptoms (both persistent and de novo) increased with age, and intermittent catheterization was significantly more common among patients in the seventh decade. The investigators concluded that while satisfaction remained high across all age groups, OAB symptom burden increased with age. Nevertheless, overall complication rates remained low, regardless of age. Schütze et al. compared 873 elderly or comorbid women treated with either TOT or retropubic TVT to evaluate the impact of age, BMI, and comorbidities on complications [20]. Postoperative complications affected 19.4% of the cohort, primarily increased post-void residual volume. Age, BMI, and comorbidities showed no significant impact on intraoperative complications; however, the TOT procedure demonstrated significantly fewer intraoperative complications (*p* = 0.001, OR: 0.281). The investigators recommended TOT for treating SUI in older, obese, and comorbid women. An Austrian multicenter survey comparing retropubic and transobturator TVT approaches in 554 women found that advancing age correlated with reduced efficacy, especially for transobturator techniques, though this effect was less pronounced than that of obesity or high parity [21]. A Swedish nationwide register study of 5200 women aged 55–94 years showed concerning trends, with cure rates declining from 88.5% in 55–64 year-olds to 64.2% in those ≥75 years [8]. However, this study included all MUS types and may have been influenced by patient selection bias and varying surgical expertise across the national database. Lo et al. investigated MUS surgical outcomes in 688 women across three age groups: young (<64 years), elderly (65–74 years), and old (>75 years), with one-year follow-up [22]. The overall objective cure rate was 88.2% and the subjective cure rate was 85.9%. Objective cure rates declined with age: 91.0% in the young, 80.6% in the elderly, and 66.7% in the old groups. Subjective cure rates showed similar trends at 89.2%, 77.6%, and 58.3%, respectively. Quality-of-life scores improved significantly across all groups, with the most significant improvement in older women. Intrinsic sphincter deficiency was significantly associated with surgical failure in older patients. The investigators concluded that MUS is safe and effective for both younger and older women, though cure rates decline with age.

Emerging technologies, such as partially absorbable mesh, may offer a viable alternative, potentially maintaining comparable success rates to conventional mesh while reducing the incidence of long-term mesh-associated complications. Partially absorbable mesh may reduce erosion because the absorbable component gradually decreases the overall foreign-body load, leading to less chronic tissue reaction and improved integration of the remaining permanent fibers. This reduced inflammatory response and lower mechanical stress on surrounding tissues are believed to minimize the risk of erosion. The present study evaluated the long-term safety and effectiveness of the Serasis^®^ transobturator MUS in women aged 65 years and older compared to a younger control group. The Serasis^®^ bi-component structure, featuring absorbable PGACL fibers that resorb within 90–120 days, and therefore may offer particular advantages in the elderly population. Results of the present study demonstrate similar favorable outcomes across age groups, with an 82.9% subjective cure rate at 4 months and 79.6% at long-term follow-up in elderly patients compared to 86.4% and 85.4% in younger patients, respectively. These results align with the higher end of published cure rates [23,24,25], potentially reflecting the technical advantages of the partially absorbable mesh system. Consistent with existing literature, results of the present study also confirm that postoperative OAB increases with age. Persistent OAB symptoms were significantly more common in elderly patients at both 4 months (18.3% vs. 9.3%, *p* = 0.02) and long-term follow-up (18.3% vs. 9.0%, *p* = 0.05). Similarly, postoperative voiding dysfunction was significantly more common in elderly patients (12.8% vs. 5.9%, *p* = 0.04), reflecting age-related changes in detrusor contractility and potentially altered tissue response to mesh placement. However, these complications were manageable and did not significantly impact overall patient satisfaction. An important observation is the low incidence of vaginal mesh erosion necessitating surgical removal, documented in 1.6% of elderly patients at long-term follow-up. This rate compares favorably with the 2–4% reported for traditional polypropylene slings in similar age groups [26,27]. This finding supports the hypothesis that the partially absorbable PGACL component may reduce long-term mesh-related complications in elderly patients who face a higher risk due to tissue fragility, impaired healing, and comorbidities. Additionally, all measured outcome parameters were comparable between the 65–74 and 75–84 year subgroups. These findings suggest that chronological age alone within the elderly range may not determine surgical success, supporting an individualized approach to patient selection based on overall health status and functional capacity rather than strict age cutoffs. Multivariate logistic regression analysis identified preoperative MUI (OR 2.1, 95% CI 1.2–3.7, *p* = 0.01), BMI > 30 kg/m^2^ (OR 1.8, 95% CI 1.1–3.0, *p* = 0.02), and concomitant stage 3 POP repair (OR 2.3, 95% CI 1.3–4.1, *p* = 0.004) as independent predictors of treatment failure. Specifically, age ≥ 65 years was not an independent predictor of failure (OR 1.3, 95% CI 0.8–2.1, *p* = 0.31). This finding reinforces that patient selection should focus on modifiable risk factors and overall health status rather than age alone.

The present study demonstrates several key strengths. Firstly, it includes a comprehensive comparative analysis of 257 elderly women aged 65 and above, further categorized into two age groups: 65–74 years (N = 208) and 75–84 years (N = 49). These are compared with a cohort of 118 younger women, providing detailed clinically relevant data for patient counseling. Secondly, the mean 8.5-year follow-up (range, 6.8–10.9 years) represents among the longest reported follow-ups for MUS studies in elderly patients and provides the first long-term data for partially absorbable mesh technology in this population. This long-term follow-up is particularly significant given concerns about late mesh-related complications and repair durability in elderly patients. Furthermore, the present study is the first to focus specifically on partially absorbable mesh technology in elderly patients, with the low erosion rates providing preliminary evidence supporting the potential benefits of this technology. Comprehensive baseline characterization, including detailed comorbidity assessment and POP staging, allows meaningful comparison with other studies and reflects real-world clinical practice. Finally, the standardized telephone follow-up methodology ensures consistent data collection while reducing loss-to-follow-up bias that might preferentially affect elderly patients with mobility limitations or cognitive decline.

This study has several limitations that should be acknowledged. First, the retrospective design introduces the possibility of selection bias, as surgical decisions were made at the discretion of the treating physician. Second, the absence of standardized frailty assessment tools restricted the characterization of functional status beyond chronological age and comorbidity count [28]. Third, recall bias may have affected the accuracy of self-reported data, particularly among older participants, where age-related memory lapses could compromise reliability. Fourth, all procedures were performed by experienced urogynecologists, which may limit the generalizability of the findings to other practice settings. Fifth, validated questionnaires for lower urinary tract symptoms were not employed; instead, symptom assessment relied on clinical evaluation, including medical history, physical examination, and objective testing. Finally, long-term outcomes were assessed through a standardized telephone survey without clinical examination. While this approach provided consistent and clinically relevant information, it may not fully capture patient-reported outcomes.

## 5. Conclusions

Results of the present study show that the Serasis^®^ partially absorbable transobturator MUS is both safe and effective in elderly women with SUI. The combination of a low complication rate and sustained long-term efficacy supports the clinical utility of this approach in this population. Notably, the use of partially absorbable mesh may offer specific advantages by potentially minimizing long-term mesh-related complications. Therefore, advanced age alone should not be considered a contraindication for MUS surgery. Clinical decisions should be individualized, taking into account symptom severity, impact on quality of life, overall health status, and patient preferences.

## Figures and Tables

**Table 1:** Baseline Demographic and Clinical Characteristics.

Mean ± SD, or N (%)	<65 Years (N = 118)	≥65 Years (N = 257)	65–74 Years (N = 208)	75–84 Years (N = 49)	*p
Age (years)	54.3 ± 5.2	71.1 ± 4.2	69.5 ± 2.8	78.2 ± 2.6	<0.001
Parity	2.8 ± 1.3	2.9 ± 1.2	2.9 ± 1.2	3.0 ± 1.1	0.42
BMI (kg/m^2^)	26.8 ± 4.2	27.2 ± 3.9	27.1 ± 3.8	27.6 ± 4.3	0.35
Comorbidities	0.8 ± 0.9	2.1 ± 1.4	2.0 ± 1.3	2.4 ± 1.6	<0.001
Prior hysterectomy	10 (8.5%)	40 (15.6%)	32 (15.4%)	8 (16.3%)	0.08
SUI duration (years)	4.2 ± 3.8	5.8 ± 4.2	5.6 ± 4.1	6.4 ± 4.5	0.001
MUI	32 (27.1%)	78 (30.4%)	63 (30.3%)	15 (30.6%)	0.54

*p—comparison between <65 years and ≥65 years groups. SUI = Stress Urinary Incontinence; MUI = Mixed Urinary Incontinence.

**Table 2:** Intraoperative and Perioperative Data.

Mean ± SD, or N (%)	<65 Years (N = 118)	≥65 Years (N = 257)	65–74 Years (N = 208)	75–84 Years (N = 49)	*p
Operative time (min)	24.8 ± 11.2	26.1 ± 12.3	25.9 ± 12.1	26.8 ± 13.2	0.31
Blood loss (mL)	28.5 ± 8.9	31.2 ± 9.8	30.8 ± 9.6	32.4 ± 10.7	0.02
Anterior colporrhaphy	65 (55.1%)	142 (55.3%)	115 (55.3%)	27 (55.1%)	0.97
Posterior colporrhaphy	58 (49.2%)	124 (48.2%)	100 (48.1%)	24 (49.0%)	0.87
Hospital stay (hours)	16.2 ± 8.4	18.9 ± 9.7	18.6 ± 9.5	20.1 ± 10.3	0.01
Same-day discharge	89 (75.4%)	168 (65.4%)	138 (66.3%)	30 (61.2%)	0.06

*p—comparison between <65 years and ≥65 years groups.

**Table 3:** Four-Month Clinical Outcomes.

N (%)	<65 Years (N = 118)	≥65 Years (N = 257)	65–74 Years (N = 208)	75–84 Years (N = 49)	*p
Objective cure	106 (89.8%)	224 (87.2%)	182 (87.5%)	42 (85.7%)	0.52
Subjective cure	102 (86.4%)	213 (82.9%)	173 (83.2%)	40 (81.6%)	0.39
Patient satisfaction (≥80)	108 (91.5%)	228 (88.7%)	185 (88.9%)	43 (87.8%)	0.42
Postoperative voiding dysfunction	7 (5.9%)	33 (12.8%)	25 (12.0%)	8 (16.3%)	0.04
Catheterization >2 days	2 (1.7%)	12 (4.7%)	9 (4.3%)	3 (6.1%)	0.15
Persistent OAB	11 (9.3%)	47 (18.3%)	37 (17.8%)	10 (20.4%)	0.02
De novo OAB	8 (6.8%)	22 (8.6%)	18 (8.7%)	4 (8.2%)	0.58

*p—comparison between <65 years and ≥65 years groups.

**Table 4:** Long-Term Clinical Outcomes.

N (%)	<65 Years (N = 89)	≥65 Years (N = 186)	65–74 Years (N = 152)	75–84 Years (N = 34)	*p
Subjective cure	76 (85.4%)	148 (79.6%)	122 (80.3%)	26 (76.5%)	0.27
Patient satisfaction ≥80	81 (91.0%)	164 (88.2%)	135 (88.8%)	29 (85.3%)	0.51
Persistent SUI	13 (14.6%)	38 (20.4%)	30 (19.7%)	8 (23.5%)	0.27
Reoperation for SUI	6 (6.7%)	16 (8.6%)	12 (7.9%)	4 (11.8%)	0.61
Mesh erosion	1 (1.1%)	3 (1.6%)	2 (1.3%)	1 (2.9%)	0.74
Chronic pelvic pain	2 (2.2%)	3 (1.6%)	2 (1.3%)	1 (2.9%)	0.71
Dyspareunia	4 (4.5%)	12 (6.5%)	10 (6.6%)	2 (5.9%)	0.54
Persistent OAB	8 (9.0%)	34 (18.3%)	27 (17.8%)	7 (20.6%)	0.05
De novo OAB	5 (5.6%)	15 (8.1%)	12 (7.9%)	3 (8.8%)	0.48

*p—comparison between <65 years and ≥65 years groups.

## Data Availability

The original contributions presented in the study are included in the article; further inquiries can be directed to the corresponding authors.

## Abbreviations

The following abbreviations are used in this manuscript:

MUS	Mid Urethral Sling
SUI	Stress Urinary Incontinence
LUTS	Lower Urinary Tract Symptoms
OAB	Overactive bladder
MUS	Mixed Urinary Incontinence
UUI	Urge Urinary Incontinence
POP	Pelvic Organ Prolapse
PAGCL	Polyglycolic Acid–Caprolactone copolymer
